# Current Molecular Therapeutic Agents and Drug Candidates for *Mycobacterium abscessus*

**DOI:** 10.3389/fphar.2021.724725

**Published:** 2021-08-30

**Authors:** Nguyen Thanh Quang, Jichan Jang

**Affiliations:** Molecular Mechanisms of Antibiotics, Division of Life Science, Department of Bio and Medical Big Data (BK21 Four Program), Research Institute of Life Science, Gyeongsang National University, Jinju, South Korea

**Keywords:** *Mycobacterium abscessus*, anti-*M. abscessus*, antibiotics, drug resistance, drug discovery

## Abstract

*Mycobacterium abscessus* has been recognised as a dreadful respiratory pathogen among the non-tuberculous mycobacteria (NTM) because of misdiagnosis, prolonged therapy with poor treatment outcomes and a high cost. This pathogen also shows extremely high antimicrobial resistance against current antibiotics, including the anti-tuberculosis agents. Therefore, current chemotherapies require a long curative period and the clinical outcomes are not satisfactory. Thus, there is an urgent need for discovering and developing novel, more effective anti-*M. abscessus* drugs. In this review, we sum the effectiveness of the current anti-*M. abscessus* drugs and drug candidates. Furthermore, we describe the shortcomings and difficulties associated with *M. abscessus* drug discovery and development.

## Introduction

A steady increase in the morbidity and mortality rates of non-tuberculous mycobacteria (NTM) has been noted worldwide; surpassing the numbers of tuberculosis (TB), NTM is becoming a new global health concern. NTM has been noted to increasingly cause pulmonary-associated morbidity and mortality in the United States, although NTM lung disease remains uncommon in the general United States population (estimated in 40 cases/100,000 persons). However, NTM is highly prevalent among adults and children with cystic fibrosis (CF). According to an analytical study in the epidemiology of pulmonary NTM sputum positivity from 16,153 patients with CF in the United States, 20% (3,211) had a pathogenic NTM species. Among the NTM, 61% had *Mycobacterium avium* complex (MAC) and 39% had *M. abscessus* (hereafter *Mab*) (Adjemian et al., 2018). Among the NTM, *Mab* is described as an opportunistic, emerging, non-tuberculous, and saprophytic *Mycobacterium* found commonly in soil and water systems (Falkinham, 2013; Morimoto et al., 2018). However, this organisms can cause nosocomial outbreaks and pseudo-outbreaks due to contaminated materials (Roux et al., 2016). For instance, NTM has been detected in hospital ice machines, water-cooling systems and haemodialysis unit water supplies (Ratnatunga et al., 2020). Furthermore, recent studies demonstrated that NTM species, including *Mab,* have been identified in showerhead biofilms, which have become the primary source of NTM exposure to humans with a high organism density (Gebert et al., 2018).

The clinical spectrum of *Mab* has been broadly categorised as a pulmonary and extrapulmonary disease. *Mab* is the most common cause of rapidly growing mycobacteria (RGM) pulmonary infections, particularly in immunocompromised patients, such as those with CF, human immunodeficiency virus-positive status, chronic obstructive pulmonary disease, and bronchiectasis (Bryant et al., 2013; Mougari et al., 2016; Skolnik et al., 2016; Wassilew et al., 2016; Andrew et al., 2019; Stephenson et al., 2019; Johansen et al., 2020b). Furthermore, according to recent studies, most *Mab* pulmonary disease cases have been identified in healthy older adults with no history of smoking but who have lung airway abnormalities (Ryan and Byrd, 2018). The pulmonary *Mab* disease is known as a chronic and incurable disease and it requires treatment with parenteral antibiotics for 2–4 months followed by long-term macrolide-based antibiotic therapy (Griffith et al., 2007). In contrast, *Mab* extrapulmonary infection can occur many sites such as skin, soft tissue, and bone infections after medical procedures or traumatic injuries (Wi, 2019). The recommended duration of therapy for extrapulmonary infection is usually a total of 4–6 months of antibiotic treatment with an initial combination of parenteral antibiotics for at least 2 weeks with a high success rate (Griffith et al., 2007).

The *Mab* group is known to consist of three subspecies, namely, *Mab* subsp. *abscessus*, *Mab* subsp. *massiliense* and *Mab* subsp. *bolletii* (Minias et al., 2020). Each subspecies is different in terms of clinical outcomes and typical antimicrobial susceptibility profile (Blauwendraat et al., 2012; Harada et al., 2012; Shin et al., 2013; Jeong et al., 2017; Abate et al., 2019). The macrolide (azithromycin and clarithromycin)-resistant ability of *Mab* subsp. *abscessus* is induced by an adaptive resistance mechanism using the inducible ribosomal methylase gene *erm* (41) (Stout and Floto, 2012; Rubio et al., 2015; Christianson et al., 2016; Abate et al., 2019). Therefore, great caution is required when using macrolide to treat *Mab* infections (Maurer et al., 2014). Although *Mab* subsp. *massiliense* is the latest subspecies in this group; it is more widely distributed than other subspecies (Sabin et al., 2017). This subspecies has more favourable clinical outcomes than the other two members because it lacks the functional *erm* gene (Koh et al., 2011; Shallom et al., 2013; Park et al., 2017; Abate et al., 2019). Recent studies have shown that the transmission source of *Mab* subsp. *massiliense* is primarily the sputum with high organism loads rather than an environmental source, and most recent outbreaks occur in transplant centres, serving patients with CF (Sabin et al., 2017). Meanwhile, *Mab* subsp. *bolletii* is the rarest among the three subspecies, and it is also resistant to clarithromycin and kanamycin (Sabin et al., 2017).

Based on the American Thoracic Society/Infectious Diseases Society of America recommendation, *Mab* therapy comprises intravenous injection of amikacin with cefoxitin or imipenem and an oral dose of macrolide (Jeon et al., 2009). However, *Mab* is resistant to many antibiotics, including the above regimen, thus making it difficult to cure. Half of all patients were cured when this regimen was used, but most cases relapsed and died (Kim et al., 2019; McNeil et al., 2020). This poor success rate is mainly caused by rapidly emerging drug resistance due to their natural and acquired multidrug resistance to antibiotics. Even first-line anti-TB drugs, such as isoniazid and rifampicin, are not active against *Mab*. This resistance mechanism presumably involves the efflux pump mechanism, drug inactivation by ADP-ribosyltransferase and erythromycin resistance genes. Thus, no antibiotic class or regimen is effective for long-term sputum smear conversion in pulmonary *Mab* infections. Novel alternative drugs to the existing regimen are therefore required. Significant efforts to develop novel anti-TB drugs have been launched, but these drugs are deemed less potent against RGM, particularly for *Mab* (Chopra et al., 2011). For instance, Telacebec (Q203), a drug candidate in phase II clinical trial for *Mycobacterium tuberculosis* (*Mtb*), targets the QcrB in cytochrome *bc*_*1*_ complex, which failed to inhibit the *Mab* growth at the 10,000-fold minimum inhibitory concentration required to inhibit the growth of 50% of *Mtb* (MIC_50_) (Sorayah et al., 2019). *Mab* drug pipelines are rarely populated and are primarily focused on the reformulation of approved antibiotics or repurposing. According to records from NIH ClinicalTrials.gov, there are only six recruiting, three completed, one terminated and one unknown clinical trial to evaluate drug efficacy. However, these clinical trials have been conducted mainly using current antibiotics in different types of drug administration, such as inhalation, new drug encapsulation with biocompatible liposomes and new drug combinations (Table 1). There are currently no FDA-approved antibiotics to treat *Mab* pulmonary disease (Maggioncalda et al., 2020). Although discovering novel drugs against *Mab* is receiving much scientific attention, our current endeavours remain insufficient. Thus, discovering new alternative compounds for *Mab* infection treatment are urgently needed. In this context, many different screens have been recently performed, such as reporter-based assays, resazurin-based microplate assay and image-based phenotypic screens (Gupta et al., 2017; Jeong et al., 2018; Richter et al., 2018; Kim et al., 2019; Malin et al., 2019; Hanh et al., 2020a, 2020b). However, there is still a poor, promising new chemical lead waiting for clinical trials and market release (Hanh et al., 2020a). This may be because of the intrinsic drug-resistant mechanism that generates the low hit rate for compounds targeting *Mab* (Malin et al., 2019). The hit rate of *Mab* screens is extremely lower than the results obtained for *Mtb* screens (Malin et al., 2019). In addition, recent *Mab* drug screens have used preselected compounds and compound libraries reconstructed using compounds with known antimycobacterial or antibacterial properties (Malin et al., 2019). Therefore, new libraries designed with expanded chemical diversity should be prepared to identify new chemical entities. Lastly, more reliable cell-based screening that can mimic the *Mab*-infected host environment is strongly required.

**TABLE 1:** Current clinical trials evaluating regimens for *Mab* treatment (ClinicalTrials.gov).

Status	Title	Treatment	Type	No. of participant	Phase	Estimated completion date
Recruiting	Liposomal amikacin inhalation in *Mab* patients	Liposomal amikacin	Observational	400		March 31, 2024
	Finding the optimal regimen for *Mab* treatment	Amikacin/Tigecycline/Imipenem/Cefoxitin/Azithromycin/Clarithromycin /Clofazimine/Ethambutol/Linezolid/co-trimoxazole/Doxycycline/Moxifloxacin/Bedaquiline/Rifabutin	Interventional	300	2 and 3	August 31, 2023
	Pilot study to assess the effect of intermittent iNO on the treatment of NTM lung infection in CF and non-CF patients	LungFit	Interventional	20	N.A.	May 2022
	Study of Mycobacterial infections		Observational	1000		January 1, 2001
	IV gallium study for patients with cystic fibrosis who have NTM	Gallium nitrate	Interventional	40	1	April 30, 2023
	The Italian registry of pulmonary Non-tuberculous Mycobacteria		Observational	500		December 31, 2022
Completed	Inhaled nitric oxide for patients with *Mab*	Nitric oxide	Interventional	9	2	April 11, 2019
	Liposomal amikacin for inhalation in the treatment of *Mab* lung disease	LAI plus multi-drug regimen	Interventional	30	2	December 31, 2019
Terminated	Trial of inhaled molgramostim in CF subjects with NTM infection	Molgramostim nebulizer solution and eFlow® Nebulizer System (PARI Pharma GmbH)	Interventional	14	2	October 2, 2020

LungFit: experimental device that produces nitric oxide from the ambient air.

INO, inhaled nitric oxide; IV, intravenous; LAI, liposomal amikacin for inhalation; NA, not applicable.

### Effectiveness and Limitation of Current Anti-Mab Drugs and Drug Candidates

Several clinical guidelines are recommended and in use (Haworth et al., 2017; Daley et al., 2020). However, these recommendations are based on a lack of high-level clinical evidence, often the clinical opinion or clinical case report (Lipman et al., 2021). *Mab* infection normally requires 18 months of long-term therapy with multidrug. The conventional regimen includes macrolide as a key drug in combination with two parenteral agents, often amikacin with a β-lactam-imipenem or cefoxitin for the initial phase (Haworth et al., 2017). This therapy should be given for at least 2–4 months, followed by oral macrolide based therapy (Victoria et al., 2021). The 2017 British Thoracic Society guidelines recommended a new regimen for the initial phase as an initial phase of at least 4 weeks course of intravenous amikacin, tigecycline, and imipenem administration with a macrolide. For the continuation phase, nebulized amikacin and an oral macrolide in combination with one to three of the following oral antibiotics guided: linezolid, clofazimine, minocycline cotrimoxazole, and moxifloxacin (Haworth et al., 2017). The main goal of this guideline is 12-month sputum culture conversion. However, recurrence and several adverse effects are frequent, making this outcome unrealistic for many patients (Lyu et al., 2011; Koh et al., 2014). Here, we present current anti-*Mab* drugs, problems and provide an update on recent developments in the *Mab* drug-development pipeline.

### Macrolide

**FIGURE 1:**
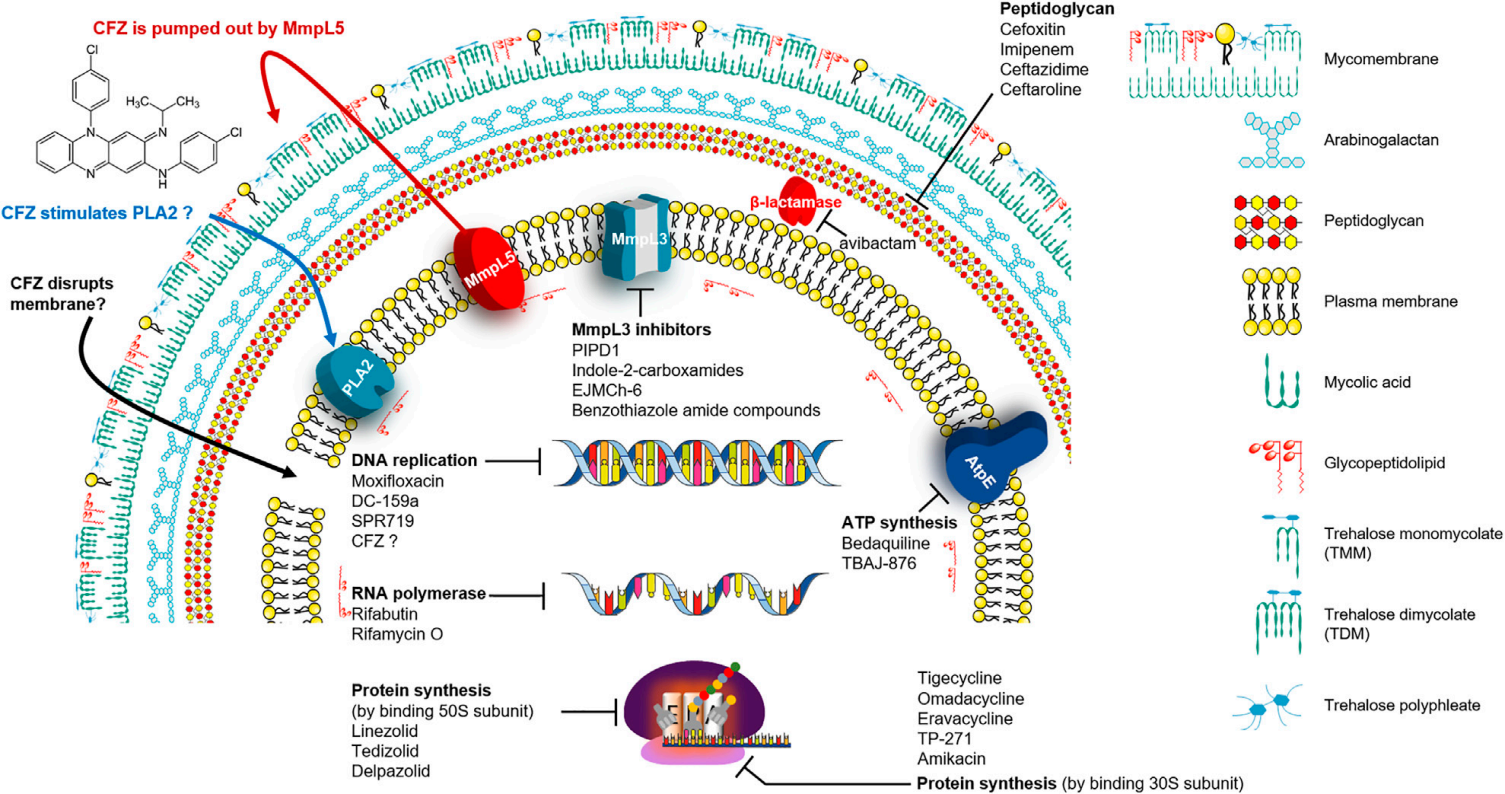
Mechanisms of action (MOA) of anti-*Mab* drugs and drug candidates.

Treatments for *Mab* infection require a long-term course with multiple antibiotics. Among them, the second-generation macrolides clarithromycin and azithromycin are cornerstone components of *Mab* treatment (Griffith et al., 2007). Although macrolides are the main agents of *Mab* multidrug therapy, the treatment success rates are poor owing to macrolide-resistant strains. The primary macrolide-acquired resistance normally occurs via a specific position on the 23S rRNA *rrl* gene; the 2058 (A2058G/C/T) or 2059 (A2059G/C) mutation in *rrl* provides resistance to clarithromycin (de Carvalho et al., 2018; Wu et al., 2018; Daniel-Wayman et al., 2019; Richard et al., 2020). Furthermore, *Mab.* subsp. *abscessus* and *Mab*. subsp. *bolletii* contain intrinsic resistance against macrolides mediated by *erm* (41). Upon exposure to the macrolide, *Mab* induces the expression of the *erm* (41) gene, and its gene product transfers methyl groups to adenine in the peptidyl region of 23S rRNA, consequently modifying the binding site of clarithromycin on the 23S ribosomal RNA (Stout and Floto, 2012; de Carvalho et al., 2018) (Figure 1). Exposure to subinhibitory concentrations of clarithromycin induces transcription of *erm* (41) by induction of a transcriptional regulator *whiB7* (Pryjma et al., 2017). However, Nash *et al.* reported some clinical isolates that contain non-functional *erm* (41). These strains did not show macrolide-inducible resistance under extended incubation with clarithromycin. Instead, the authors revealed that the strains comprise loss-of-function on the *erm* (41) gene with a T-to-C substitution at position 28 (C28 sequevar), resulting in an amino acid substitution from Trp to Arg at codon 10 (Nash et al., 2009; Brown-Elliott et al., 2015). Therefore, it would be worthy to sequence the *erm* (41) gene for prediction of macrolide susceptibility. In contrast, isolates of *Mab* subsp. *massiliense* carry a non-functional *erm* (41) gene that contains 397 bp deletion, including position 28T, consequently do not show inducible-macrolide resistance, albeit it showed macrolide resistance due to *rrl* mutants (Brown-Elliott et al., 2015).

To overcome the existing macrolide resistance mechanisms, new drug discovery and drug design have been evaluated. For instance, a novel class of macrolide antibiotics named “macrolones” comprising a macrocyclic moiety, linker, and either free or esterified quinolone group showed excellent antibacterial activity towards erythromycin-resistant gram-positive and gram-negative bacterial strains (Čipčić Paljetak et al., 2016). Furthermore, the macrolones possess a low clearance, large volume of distribution, long half-life and possess favourable pharmacokinetic properties by accumulating in inflammatory cells, consequently complying with a once-daily dosing potential (Munić Kos et al., 2013). However, although there are considerable data available on its *in vitro* activity, mode of action, *in vivo* efficacy and its recognition as a superior compound than other known macrolides, more detailed information on the structure-associated binding mode to the ribosome is needed (Jelić and Antolović, 2016). Furthermore, its activity against *Mab* is yet to be determined. A better understanding of the macrolones in various animal models and how the macrolones in *Mab* impact potency is also needed.

### β-Lactams and β-Lactamase Inhibitor

β-lactams are a widely-used antibiotic class, and their safety and efficacy profiles have been well-documented. β-lactams inhibit the synthesis of an essential component of the bacterial cell wall, the peptidoglycan (Hartmann et al., 1972). Peptidoglycan of the *Mab* contains predominantly 3→3 cross-links (64–74%) generated by L,D-transpeptidases that are considered attractive targets for anti-*Mab* drugs. (Lavollay et al., 2011). Penicillin-binding proteins (PBPs) bind to β-lactams and this binding, in turn, interrupts the terminal transpeptidation process; consequently, it induces loss of viability and lysis of bacterial cells (Eckburg et al., 2019) (Figure 1). These have been studied extensively to treat drug-resistant *Mtb* infections, and certain β-lactam subclasses also exhibit activity against *Mab*. However, most of the other β-lactam antibiotics are not considered owing to their rapid hydrolysis by broad-spectrum β-lactamase (MAB_2875; BlaMab), which was reported as the major determinant of β-lactam resistance in *Mab* (Dubée et al., 2014; Soroka et al., 2014; Pandey et al., 2019). Only two β-lactams, such as cefoxitin and imipenem, which are relatively stable in the presence of BlaMab, showed moderate *in vitro* activities, and both compounds also exhibited a favourable *in vivo* activity in the *Mab*-infected zebrafish and mouse model (Table 2) (Bernut et al., 2014; Lerat et al., 2014; Moigne et al., 2020). Both drugs are currently recommended in treatment guidelines (Kozikowski et al., 2017; Pandey et al., 2019).

**TABLE 2:** The current state of *Mab* antibiotics, with compounds currently going through development. * Indicates MIC_50_ value.

Class	Name	Chemical structure	MIC_90_ (mg/L)	Efficacy
Tetracycline	Tigecycline	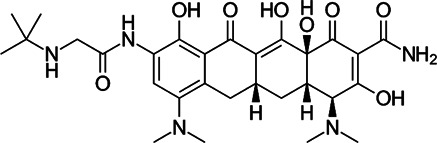	2–16	• Increasing lifespan of *Mab*-infected fruit fly
				• Proving dose-dependently effective against *Mab* in GM-CSF knockout mice
				• Combination therapy showed treatment success in retrospective analysis
				• Severe adverse effect mainly gastrointestinal distress
	Omadacycline	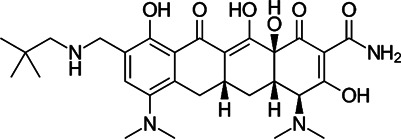	2	• Tolerability is acceptable for patients with *Mab* disease
				• Improved AUC/MIC than tigecycline (8–10 times)
				• Significantly less gastrointestinal distress and fewer TEAEs
	Eravacycline	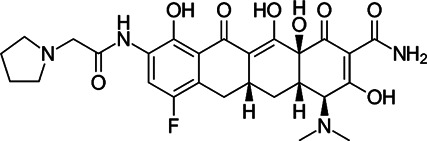	1	• Improved AUC/MIC than tigecycline (2 times)
	TP-271	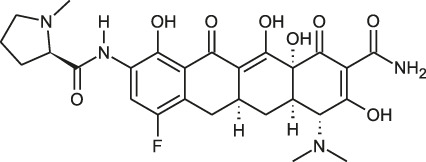	0.06–0.5	• In vivo efficacy has not yet tested against *Mab* infected animal
				• Currently under investigation in clinical phase I
Rifamycin	Rifabutin	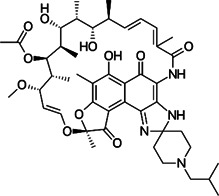	0.9–4.2	• Good intracellular penetration/distribution
				• Less drug-drug interaction
				• No antagonistic effect with anti-*Mab* antibiotics
				• Showing very good in vivo efficacy against Mab either in a zebrafish model and in NOD.CB17-Prkdcscid/NCrCrl mouse model
	Rifamycin O	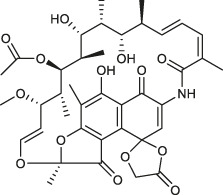	3–4.7*	• Good anti-*Mab* activity in zebrafish model of infection
Oxazolidinone	Tedizolid	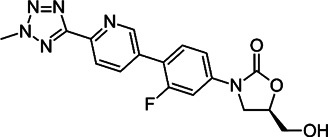	4–8	• 2–16 fold greater in vitro activity than linezolid against *Mab* Bacteriostatic activity
	Delpazolid	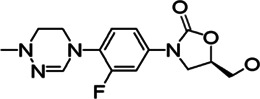	1.2*	• Comparable efficacy to linezolid in mouse model
				• Less myelosuppression in phase I clinical trial
				• Currently in clinical phase II for pulmonary TB
Diarylquinoline	Bedaquiline	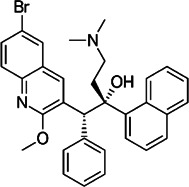	0.062–0.125	• Good in vivo efficacy in zebrafish with less abscess and cord
				• Significant bacterial reduction in GKO and SCID mouse model
				• Less effectiveness in nude mouse model
				• Doubtful result in small scale salvage therapy (4 patients) → only one patient showed improvement of clinical symptoms
	TBAJ-876	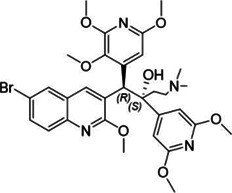	0.2–0.7	• Improved tolerability and PK profiles than bedaquiline
				• Good in vivo efficacy in mouse infection model
	Moxifloxacin	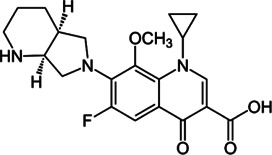	> 8	• Inducible mutational resistance
				• Maybe used as oral drug in combination with other anti-*Mab* drug
				• No mutation on *gyrA* and *gyrB* has been identified in *Mab*
Fluoroquinolones	DC-159a	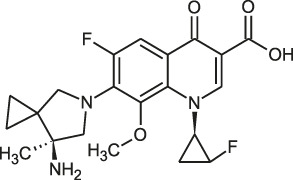	16	• 4- to 8-fold lower MIC than other quinolones to *Mab*
	Clofazimine	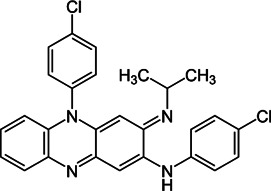	≤1	• Synergistic effect with AMK and CLA in in vitro combination
				• Long half-life, slow metabolic elimination, high conc. in macrophage MOA is not clear
				• Good in vivo efficacy in fruit fly, SCID, GKO, and GMCSF mouse
				• CFZ containing regimen was revealed as effective against *Mab* in retrospective study
	Cefoxitin	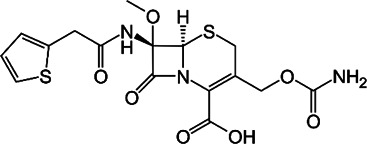	32–64	• Moderate in vitro activity
				• Good in vivo efficacy in GKO mouse model of *Mab* infection
	Imipenem	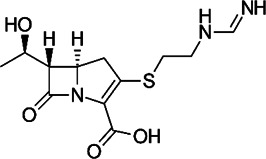	16–64	• Moderate in vitro activity
				• Good anti-*Mab* activity in zebrafish model of infection
				• Favorable in vivo efficacy in C3HeB/FeJ mouse model of *Mab* infection
				• Synergistic activity with ceftazidime (in vitro and macrophage infection model)
β-lactams	Ceftazidime	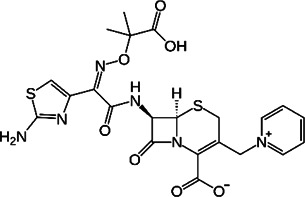	32–1024	• Poor in vitro activity
				• Synergistic activity with ceftaroline or imipenem (in vitro and macrophage infection model)
	Ceftaroline	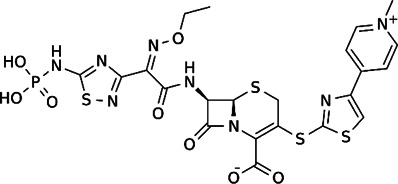	2–128	• Moderate in vitro activity
				• Synergistic activity with ceftazidime (in vitro and macrophage infection model)
β-lactamase inhibitor	Avibactam	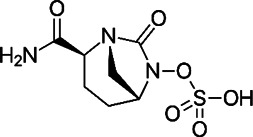		• No activity against *Mab*
				• A non-β-lactam β-lactamase inhibitor, efficiently inhibits BlaMab.
				• β-lactam combinations plus avibactam exhibited synergistic effect
				• Those combination showed significant CFU reductions in the lungs of mice
	PIPD1	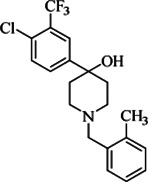	0.125–0.5	• Excellent activity in *Mab*-zebrafish infection model
				• Resistant mutation was identified on MAB_4508
	Indole-2-carboxamides	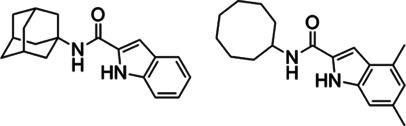	0.25 (compound 5) 0.063 (compound 25)	• ICs showed statically significant reduced bacterial load in acute SCID mouse model
MmpL3 inhibitors	EJMCh-6	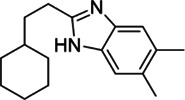	0.031–1	• Very good activity against intracellular *Mab*
				• Significant activity in zebrafish model of *Mab* infection
	Benzothiazole amides	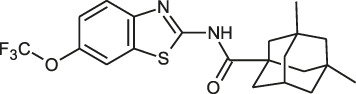	0.5 (CRS400226)	• 0.64 log_10_ bacterial load reduction with minor airway-centric inflammation in GM-CSF KO mouse
	Epetraborole	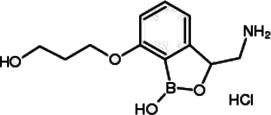	0.1–1.35	• Significant activity against *Mab* in vitro, intracellular and in zebrafish infection model
				• Clinical phase II study for UTI and intra-abdominal infection was terminated due to rapi d emergence of resistant mutant
	AR-12	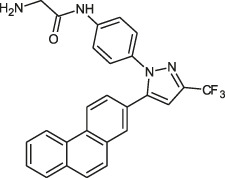	4-8	• A phase I clinical trial has been completed
				• AR-12 has received recently FDA IND-approval for cancer treatment
				• Orphan drug status in Europe for selected indications
				• Anti-pathogenic activity against bacteria, fungi, and viruses
				• Very good in vivo efficacy in *Mab* infected mouse model
New anti-*Mab* inhibitors	SPR719	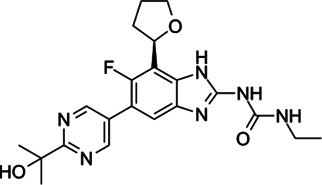	2–4	• Showed good in vivo efficacy result in *Mab. subsp. bolletii* infected SCID mouse
	Etamycin	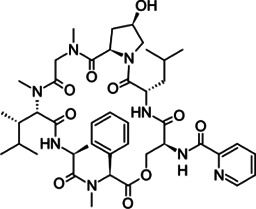	3.8–24.9	• Inhibit growth of intracellular *Mab*
				• Good activity in *Mab*-zebrafish infection model
	Thiostrepton	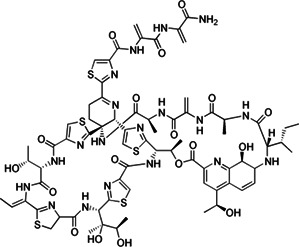	1.4–7.7	• Significant inhibition of *Mab* growth in vitro and in macrophages
				• Treated macrophages decreased proinflammatory cytokine production
				• Good activity in *Mab*-zebrafish infection model

Interestingly, recent research on avibactam, a non-β-lactam β-lactamase inhibitor combined with β-lactam antibiotics in *Mab,* has brought significant attention back to the re-use of β-lactams for *Mab* treatment. Treatment with avibactam clearly showed reduced MICs of several β-lactams against *Mab* (Kaushik et al., 2017; Eckburg et al., 2019; Story-Roller et al., 2019). Furthermore, Dubée et al., demonstrated that knockout of the β-lactam-modifying gene (MAB_2875) restored the activity of β-lactams. Furthermore, BlaMab is inactivated by the β-lactamase inhibitor avibactam, resulting in significantly improved intramacrophagic and *in vivo* activity in a zebrafish model of *Mab* infection (Dubée et al., 2014; Lefebvre et al., 2017). Recently, the use of avibactam has gained more interest (Figure 1). The triple combination of rifabutin, imipenem and avibactam achieved five-fold killing in the numbers of intracellular survival of *Mab* (Le Run et al., 2018). Therefore, inhibition of functional β-lactamase activity would be crucial for the β-lactam-repurposing strategy following *Mab* treatment. In addition, an interesting result was also reported by Pandey et al. They discovered that dual β-lactam combinations, such as that of ceftazidime with ceftaroline or ceftazidime with imipenem, showed a persistent bactericidal effect *in vitro*, and these combinations also showed dramatically reduced bacterial burden to near baseline levels of infection against *Mab*-infected THP-1 human macrophages. Again, these unexpected synergistic activities provide the possibility of re-use of a large family of β-lactam drugs as a treatment strategy against *Mab* infections through proper combinations (Pandey et al., 2019).

### Tetracycline Antibiotics

Tetracyclines were discovered in 1945; they were known to inhibit bacterial growth by blocking the attachment of charged aminoacyl-tRNA to the A site on ribosomes, inducing failure of protein synthesis (Obrecht et al., 2011) (Figure 1). Usually, the changes of tetracycline efflux or ribosomal protection become resistant to tetracycline (Greer, 2006). For this reason, several tetracycline analogues have been designed and synthesised to prevent the development of resistance to tetracyclines. Although *Mycobacterium smegmatis* and *Mtb* showed low levels of intrinsic resistance to tetracycline, *Mab* is approximately 500-fold more resistant to tetracyclines than *M. smegmatis* and *Mtb*, making them unavailable for treatment (Rudra et al., 2018).

Tigecycline, the first commercially available glycylcycline, was created to bypass the critical mechanisms of bacterial resistance to tetracyclines. Changes in the tetracycline structure allow tigecycline to show good activity against tetracycline-resistant pathogens, such as penicillin-resistant *Streptococcus pneumoniae*, methicillin-resistant *Staphylococcus aureus* (MRSA) and *Staphylococcus epidermidis* (MRSE), and vancomycin-resistant enterococcus (VRE) species. Furthermore, tigecycline is a poor substrate of the MabTetX (MAB_1496c), tetracycline-inactivating monooxygenase, and it remains more effective than other tetracyclines analogues (Johansen et al., 2020b). Tigecycline was approved in Europe and the United States to treat complicated skin diseases and complicated intra-abdominal infections as the first drug in this class of antimicrobials (Greer, 2006). Tigecycline has *in vitro* activity against the *Mab* complex, and the minimum inhibitory concentration required to inhibit the growth of 90% of the organisms (MIC_90_) was 2–16 mg/L (Singh et al., 2014). In the *Drosophila* infection model, the lifespan of *Mab*-infected *Drosophila* treated with tigecycline is much-expanded than that of *Mab*-infected *Drosophila* treated with linezolid, amikacin and cefoxitin. The combination of tigecycline and linezolid has an excellent *in vivo* efficacy in boosting infected fly survival and reducing *Mab* dissemination compared with a cocktail regimen (clarithromycin, amikacin plus cefoxitin) (Oh et al., 2014). Furthermore, inhaled tigecycline was effective against *Mab* in a dose-dependent manner in granulocyte-macrophage colony-stimulating factor (GM-CSF) knockout mice (Pearce et al., 2020). The most extensive clinical trials were conducted for 52 patients with *Mab* and *M. chelonae* infections to monitor the effect of tigecycline on patients. Tigecycline-containing regimens were used as a salvage treatment for patients with underlying CF without prior antibiotic therapy. In this study, intravenous (IV) tigecycline given (50 mg daily) for ≥1 month as part of a multidrug regimen resulted in a clinical remission rate of more than 60%*.* However, adverse events were also reported in more than 90% of the cases. The general adverse effects were vomiting and nausea (Wallace et al., 2014). Furthermore, a retrospective analysis was also performed to evaluate the *in vivo* efficacy and adverse effects of tigecycline administration, wherein 32 of the 53 patients with *Mab* pulmonary disease (60%) met the criteria for treatment success, 19 (49%) of the 39 patients with *Mab* subsp. *abscessus* pulmonary disease met the criteria for treatment success and 13 (93%) of the 14 patients with *Mab* subsp. *massiliense* pulmonary disease met the criteria for treatment success. However, tigecycline treatment has also resulted in severe adverse effects, mainly gastrointestinal distress, such as nausea and vomiting (in 29 of 60 cases; 48.3%) (Chen et al., 2019). Thus, intravenous administration of tigecycline is deemed undesirable for long-term treatment of patients (Kaushik et al., 2019). Furthermore, in 2010 and 2013, the US FDA reported an increased risk of mortality associated with tigecycline use. Therefore, a new version of tigecycline with similar or better efficacy and fewer adverse effects, preferably with oral bioavailability, is desperately needed to improve the treatment for *Mab* infections (Kaushik et al., 2019).

For these reasons, two newly developed tigecycline analogues, omadacycline and eravacycline, have been reported to show therapeutic potential. Omadacycline has an *in vitro* activity against the wild-type and drug-resistant *Mab* with an MIC_90_ 2 mg/L near the MIC of tigecycline. However, eravacycline showed half *in vitro* MIC_90_ (1 mg/L) than tigecycline. Considering the steady-state area under the curve (AUC) and MICs obtained against *Mab*, the free drug AUC/MIC ratios for omadacycline and eravacycline, given intravenously, are expected to be approximately eight to ten times higher and twice higher than tigecycline, respectively. This improved the intravenously administered pharmacokinetic/pharmacodynamic parameters, and activity data suggest that eravacycline and omadacycline could be more effective than tigecycline clinically (Kaushik et al., 2019). Recently, oral (PO) and IV formulations of eravacycline (brand name Xerava) were clinically examined, but only the IV formulation was FDA-approved for treating complicated cIAIs in patients who are 18 years or older. Eravacycline’s mechanism of action is similar to tetracycline because it blocks protein synthesis by binding to ribosomes (Lee and Burton, 2019). Omadacycline is associated with significantly less nausea and fewer treatment-emergent adverse events than tigecycline (Gotfried et al., 2017). Recently, a clinical study was reported for using omadacycline on four patients with culture-positive *Mab* disease (two patients had a cutaneous disease, one had the pulmonary disease and another had osteomyelitis and bacteraemia). In this report, the patients were treated with an omadacycline regimen, including other antimicrobial agents, for a median duration of 166 days. Omadacycline-containing regimens showed a clinical cure in three of the four patients. Omadacycline is relatively well tolerable during long-term treatment, although one patient discontinued therapy at the sixth month because of nausea. Despite this positive case report, further experiments are needed to determine the role of omadacycline in treating *Mab* disease (Pearson et al., 2020).

TP-271 is a synthetic fluorocycline antibiotic belonging to tetracyclines. Like other fluorocyclines, TP-271 inhibits protein synthesis by acting on the 30S ribosomal subunit. TP-271 showed *in vitro* activity against *Mab* with an MIC_90_ range of 0.06–0.5 mg/L, although this compound has not been tested for its *in vivo* efficacy in animal models with *Mab* infection. TP-271 has demonstrated broad-spectrum *in vitro* and *in vivo* activities against various community-acquired organisms, including *Acinetobacter baumannii*, *Staphylococcus* spp., *Streptococcus* spp., *Legionella pneumophila*, *Haemophilus influenzae*, *Moraxella catarrhalis and L. pneumophila*, and other biothreat pathogens (Cynamon et al., 2012). Furthermore, TP-271 also showed potent *in vivo* efficacy in mouse and nonhuman primate models of inhalational *Francisella tularensis* and *Bacillus anthracis* (Grossman et al., 2017a; Grossman et al., 2017b). TP-271 is currently in phase I clinical trial, in which the safety and exposure in healthy volunteers receiving TP-271 intravenously and orally is being evaluated (Grossman et al., 2017b). Therefore, TP-271 is worth evaluating for *in vivo* efficacy against inhaled *Mab* in animal models.

### Rifamycin

Rifamycins were first isolated in 1957 from *Streptomyces mediterranei*, which was later re-named *Amycolatopsis mediterranei* (Sensi et al., 1959; Verma et al., 2011). Further studies showed that although rifamycin B was microbiologically inactive, its activity depended on its transformation into an active product in aqueous solutions, such as test cultures or body fluids. The transformed products, rifamycin O, SV and S, were then isolated and are microbiologically active. Of these, rifamycin SV had the best *in vivo* activity, tolerability and solubility; it has been used to treat gram-positive bacterial infections in many countries. However, rifamycin SV has poor anti-TB activity (Banerjee et al., 1992). Rifampicin targets the β-subunit of bacterial RNA polymerase (RpoB; Rv0667) in *Mtb* (Rominski et al., 2017; Ganapathy et al., 2019). In this context, rifampicin resistance is primarily caused by *rv0667* mutation as acquired drug resistance. Critically, rifampicin retains bactericidal activity against intra-macrophages and slow metabolising *Mtb,* such as drug-tolerant and non-replicating bacteria. Furthermore, rifamycins can sterilise mycobacteria in caseum, the necrotic material at the centre of granulomas, which is difficult to eradicate. Thus, rifampicin showed strong potency against *Mtb in vivo* and reduced relapse rate, consequently shortening TB therapy to 6 months (Ganapathy et al., 2019) (Figure 1).

However, rifampicin is excluded from the treatment of *Mab* lung disease owing to its low potency. It showed a high *in vitro* MIC_90_ value against all *Mab* subspecies in cation-adjusted Mueller–Hinton broth (approximately 165 mg/L) (Aziz et al., 2017). As rifampicin does not cause acquired resistance by *rpoB* mutation in *Mab*, its resistance by *Mab* is attributed to an intrinsic mechanism. Recently, Rominski et al. showed that rifampicin ADP-ribosyltransferase (MAB_0591; Arr_Mab), which catalyses ADP-ribosylation at the C23 position of rifamycin, is a significant innate rifamycin resistance in *Mab* subsp. *abscessus* ATCC 19977 via gene knockout studies. Deletion of MAB_0591 improved the potency of rifamycin compared with the *Mab* parental type strain and the rifamycin MIC was increased when mutant was complemented with MAB_0591. Thus, Arr_Mab is the major innate rifamycin resistance determinant of *Mab* (Rominski et al., 2017).

Also, the *Mab* genome encodes many proteins, such as members of the major facilitator family, ATP-banding cassette transporters and MmpL proteins, which may be involved in drug efflux systems. It also encodes a small multidrug-resistant family, a family of lipophilic drug efflux proteins and a multidrug-resistant *stp* protein involved in spectinomycin and tetracycline resistance similar to *Mtb* (Nessar et al., 2012; Sassi and Drancourt, 2014).

However, rifamycin has been recently used to manage *Mab*. Aziz *et al.* conducted *in vitro* screening using a collection comprising 2,662 US FDA-approved compounds and narrowed down rifabutin as a hit. This rifamycin analogue had an MIC_90_ of 2.5 mg/L against *Mab* bamboo strain, three *Mab* subspecies and clinical isolates (Aziz et al., 2017). In this context, rifabutin has been spotlighted recently. Furthermore, rifabutin showed *in vivo* efficacy against *Mab* in a zebrafish model, extending the lifespan of the *Mab*-infected zebrafish and in NOD.CB17-Prkdcscid/NCrCrl mouse model compared with clarithromycin (Johansen et al., 2020a; Dick et al., 2020). Rifabutin treatment applications have some benefits, namely high intracellular penetration, high volume of distribution, adequate concentrations at the infection site, less drug–drug interaction and better toleration by a large proportion of patients. Furthermore, there are no antagonistic effects with other clinically used anti-*Mab* antibiotics that have not been reported (Ganapathy et al., 2019). In addition, the chemical structures of rifabutin are different from other rifamycin analogues. It lacks a hydroquinone moiety that *Mab* readily metabolises at the C1 and C4 positions. Thus, this structural difference is considered a key factor for anti-*Mab* activity (Hanh et al., 2020b). Nonetheless, rifabutin is also a substrate of Arr, a member of rifamycin; consequently, its activity is inhibited by Arr. Thus, co-treatment with Arr-inhibitor or structural modification of rifabutin might improve its potency against *Mab* (Schäfle et al., 2021). Recently, another rifamycin analogue, i.e., rifamycin O, which lacks hydroquinone in the C1 and C4 positions, also showed anti-*Mab* activity in an infected zebrafish model (Hanh et al., 2020b).

### Oxazolidinone

Oxazolidinone is determined to have bactericidal activity against many gram-positive bacteria, such as vancomycin-intermediate strains, VRE, MRSA and penicillin-resistant pneumococci. Linezolid is the first oxazolidinone to be developed; it exhibits a high degree of *in vitro* activity against various gram-positive pathogens. It also inhibits bacterial growth by binding to a site on the bacterium’s 50S subunit 23S ribosomal RNA (Figure 1). This binding prevents 70S ribosomal unit formation; consequently, protein synthesis is inhibited (Rosati, 2017; Cho and Jang, 2020). Furthermore, linezolid exhibits bactericidal activity against *Mtb* and has been used to treat rifampicin-resistant and multidrug-resistant TB. However, prolonged administration is often limited by long-term side effects, such as reversible myelosuppression, potentially irreversible optic and peripheral neuropathies (Cho and Jang, 2020). Linezolid has shown a weak *in vitro* activity against *Mab* infections with a modal MIC of 32 mg/L and an MIC_90_ of 64 mg/L (Wallace et al., 2001).

Compared with linezolid, tedizolid is a next-generation oxazolidinone with a favourable toxicity profile and superior penetration into the epithelial lining fluid. Its MIC_50_ and MIC_90_ were 1 and 4–8 mg/L, respectively, across all *Mab*-tested strains, and the values were 2–16-fold lower than those of the linezolid. The superior *in vitro* potency of tedizolid against *Mab* suggests that it is a potential treatment agent for *Mab* infections (Brown-Elliott and Wallace, 2017). Furthermore, pre-exposure of *Mab* complex to tedizolid sub-MICs did not initiate any drug-inducible drug resistance. Time-kill kinetics assays demonstrated the bacteriostatic activity of tedizolid against all *Mab* subspecies, even at high drug concentrations (four to eight times of the MIC) (Tang et al., 2018).

A novel oxazolidinone, delpazolid (code no. LCB01-0371) that contains cyclic amidrazone was developed by LegoChem Biosciences and showed improved safety, tolerability and pharmacokinetics (PK). It has been reported that delpazolid does not cause adverse events, such as myelosuppression, which is a severe side effect of linezolid, even after 3 weeks of repeated dosing in phase 1 clinical trial (Cho and Jang, 2020). Delpazolid has a potential broad-spectrum *in vitro* activity against *Mab* ATCC 19977 with MIC_50_ of 1.2 mg/L. Furthermore, it resulted in reduced bacterial load in the lungs to approximately 3.7 log_10_ CFUs compare to the efficacy of linezolid at 100 mg/kg. Currently, delpazolid is in phase II clinical trials for pulmonary TB. Thus, delpazolid may be a promising new class of oxazolidinones with enhanced protection that could eventually take the place of linezolid in the long-term treatment of *Mab* (Kim et al., 2017). Recently, Wen et al. evaluated the *in vitro* susceptibility of 115 isolates and 32 reference strains that are members of different RGM species against four oxazolidinones, namely, delpazolid, sutezolid, tedizolid and linezolid. The results showed that tedizolid had the most potent inhibitory activity (MIC_50_ = 1 mg/L and MIC_90_ = 2 mg/L) against *Mab in vitro*. Simultaneously, delpazolid presented the best activity against *Mycobacterium fortuitum*, giving important insights into the potential clinical application of oxazolidinones to treat RGM infections (Wen et al., 2021). However, the use of oxazolidinone in clinical infection is not simply considered by its MIC value. Thus, PK profiles and safety issues should be compared together with *in vivo* infection models. However, there is no exact comparison of PK profiles, safety issues and *in vivo* efficacy between tedizolid and delpazolid. Delpazolid received an FDA orphan drug designation, a qualified infectious disease product designation, and was selected as a fast-track target drug (Cho and Jang, 2020).

### Diarylquinoline

Bedaquiline is a diarylquinoline antibiotic that inhibits the proton pump of mycobacterial ATP synthase, resulting in ATP depletion, unstable pH homeostasis and cell death (Andries et al., 2005; Koul et al., 2007) (Figure 1). It was authorised by the FDA and the European Medicines Agency in December 2012 to treat multidrug-resistant TB (MDR-TB) (Olaru et al., 2015). *In vitro* MIC range of bedaquiline against *Mab* subspecies clinical isolates is 0.007–1 mg/L, with an MIC_50_ and MIC_90_ of 0.06 and 0.12 mg/L, respectively (Brown-Elliott and Wallace, 2017). Furthermore, verapamil, a calcium channel antagonist recognised to inhibit bacterial efflux pumps, has been tested with bedaquiline to potentiate the activity of bedaquiline against *Mab*. In the study, Viljoen *et al.*, reported that verapamil increased the efficacy of bedaquiline against *Mab* clinical isolates and low-level resistant strains, both *in vitro* and a THP-1 macrophage infection model. Thus, the authors suggested that combining efflux pump inhibitors, such as verapamil and bedaquiline, may have clinical potential as adjunctive therapy (Viljoen et al., 2019). Bedaquiline has shown various *in vivo* activity against *Mab*-infected animal models, such as zebrafish and immunocompromised mice. Research by Dupont et al. has indicated that bedaquiline has a strong protective impact on infected zebrafish larvae from *Mab*-induced killing with a reduced number of abscesses and cords (Dupont et al., 2017).

Furthermore, Obregón-Henao et al., first tested the efficacy of bedaquiline using acute GKO and severe-combined immunodeficient (SCID) mouse treatment model (Obregón-Henao et al., 2015). In the models, bedaquiline (30 mg/kg) significantly reduced bacterial loads in the lungs, spleens and livers 15 days after treatment. However, by contrast, another study demonstrated that bedaquiline (25 mg/kg) did not modify the decrease in bacterial burden (less than 1 log_10_ CFU) during the 2 months when its activity was evaluated in a nude mouse, that is, athymic mice with depletion of T cells (Lerat et al., 2014). Thus, bedaquiline efficacy in the *in vivo* animal model is uncertain and can depend on the animal model. However, recently Le Moigne et al., demonstrated that treatment with the bedaquiline plus imipenem combination enhanced *Mab* clearance rather than antibiotic treatment alone in C3HeB/FeJ mice model. Thus, studies showed that the activity of bedaquiline can be potentiated imipenem activity in combination (Moigne et al., 2020).

In 2015, Philley et al. reported the preliminary results of bedaquiline as a salvage therapy for patients with *Mab* lung disease. It had a small-scale off-label use of bedaquiline for treatment failure of lung diseases caused by *Mab*. In the study, bedaquiline was used to treat four patients with nodular and cavity radiographic features with *Mab* disease. It showed clinical improvement in all cases after 3 months with bedaquiline-containing regimen treatment and without severe side effects. However, only one patient reported improved clinical symptoms after 6 months of observation (Philley et al., 2015). Thus, there is currently no clinical evidence to show that bedaquiline is a potential option for treating *Mab* infection. Although bedaquiline shows significant clinical activity, it might not be used for long-term *Mab* treatment since it is highly lipophilic, shows a long terminal half-life and has a cardiotoxicity liability associated with QT interval prolongation (Sarathy et al., 2020).

Recently, TBAJ-876, a less lipophilic bedaquiline analogue, has a higher clearance and a lower cardiotoxic potential; also, it has been evaluated for *Mab*. TBAJ-876 displayed submicromolar *in vitro* activity (MIC_90_ ranged 0.2–0.7 mg/L) against *Mab* reference strains, including three subspecies of *Mab* and clinical isolates similar to bedaquiline. Furthermore, TBAJ-876 showed similar *in vivo* efficacy to bedaquiline at 10 mg/kg. It reduced the bacterial burden of *Mab* at 30 mg/kg compared with bedaquiline (20 mg/kg) in a *Mab*-infected mouse model (Sarathy et al., 2020). This demonstrated that TBAJ-876, with improved tolerability and PK profiles, may clinically aid in the treatment of Mab lung disease.

### Fluoroquinolones

Fluoroquinolones are used as second-line drugs for MDR-TB, and it inhibits the supercoiling action of DNA gyrase, a unique target of fluoroquinolones (Figure 1). The fluoroquinolone antibiotics include ciprofloxacin, gemifloxacin, levofloxacin, moxifloxacin and ofloxacin. Fluoroquinolones are recommended for treating macrolide-resistant *Mab* lung disease based on drug susceptibility testing results. *In vitro*, ciprofloxacin and moxifloxacin have showed high activities, with 57 and 73% susceptibility for *Mab* isolates, respectively (Park et al., 2008). However, *in vitro* MIC value is high. MICs of moxifloxacin for *Mab* complex range from 2 to more than 8 mg/L (MIC_50_ of 8 mg/L and MIC_90_ of more than 8 mg/L) (Hatakeyama et al., 2017). Although fluoroquinolone cannot be used clinically as monotherapy because of its inducible mutational resistance, both ciprofloxacin and moxifloxacin may be used as alternative oral agents for treating *Mab* lung disease combined with other drugs (Park et al., 2008). Since GyrB quinolone-resistance determining region (QRDR) confers resistance to fluoroquinolones in *Mtb*, *Mab* was also expected to comprise amino acid changes within the GyrA QRDR (Nessar et al., 2012). However, recently, Kim *et al.* reported that amino acid substitutions associated with fluoroquinolone resistance were unidentified in any of the *Mab* gyrase genes (*gyrA* and *B*) from moxifloxacin susceptible, intermediate and resistant strains (22 *Mab* subsp. *abscessus* and 24 *Mab* subsp*. massiliense*) (Kim et al., 2018). Therefore, Kim *et al.* explained that there is no clear correlated evidence between mutation of the DNA gyrase genes and moxifloxacin resistance in *Mab*, indicating that alternative mechanisms might be involved in moxifloxacin resistance, such as efflux pumps, which is well known in *Mtb* (Kim et al., 2018).

DC-159a is a newly synthesised broad-spectrum 8-methoxy-fluoroquinolone and has shown bactericidal activities against various respiratory pathogens, including multidrug-resistant *Streptococcus pneumoniae* and quinolone-resistant strains (Clark et al., 2008; Hoshino et al., 2008). Furthermore, DC-159a showed potent *in vitro* activity against quinolone-resistant multidrug-resistant *Mtb* (MIC_90_, 0.5 mg/L) and drug-susceptible isolates (MIC_90_, 0.06 mg/L). In the *in vitro* activity comparison with *Mtb*, DC-159a was 4–32-fold more potent than other quinolones (Disratthakit and Doi, 2010). DC-159a also showed the highest activities against NTM, such as *M. fortuitum*, MAC, *M. chelonae* and *M. abscessus.* The MIC range of DC-159a against *Mab* was 4–32 mg/L, which is 2–4-fold lower than those of other tested quinolones, such as moxifloxacin, levofloxacin and gatifloxacin (Disratthakit and Doi, 2010). However, its activity is unsatisfactory compared with other anti-*Mab* candidates because the *in vitro* MIC value is higher than others. Still, there is no *in vivo* efficacy data for *Mab* (Soni et al., 2016).

### Clofazimine

Clofazimine (CFZ) belongs to the riminophenazine group and has demonstrated an impressive activity against rapid-growing *Mycobacterium* (*Mab*, *M. fortuitum and M. smegmatis*) and slow-growing *Mycobacterium* (*Mtb*, MAC and *M. leprae*) (Cholo et al., 2017). Most *Mab* clinical isolates had CFZ MICs of ≤1 mg/L (Wu et al., 2018). Recently, CFZ has been used for *Mab* treatment (Martiniano et al., 2017; Yang et al., 2017). Furthermore, CFZ has shown a synergistic effect with other antimicrobial agents, including amikacin and clarithromycin, against *M. avium* and *Mab in vitro*. CFZ has several advantages as an effective drug because it has a long half-life, slow metabolic elimination, low cost, high concentration in macrophages and rapid localisation within phagocytes (Shen et al., 2010). However, CFZ has no exact mechanism of action (MOA), and information on its resistance in patients is yet undetermined (Figure 1). Although the exact MOA for CFZ is not fully understood, the cell membrane seems to be the primary action site. It has been demonstrated that CFZ is a prodrug and can disrupt bacterial membrane via interaction with intracellular redox cycling, releasing reactive oxygen species (Yano et al., 2011). Furthermore, another putative MOA is that CFZ stimulates phospholipase A2 (PLA2) activity, resulting in an accumulation of detergent-like lysophospholipids and disrupting fundamental cellular functions. Also, it has been hypothesised that it inhibits cell replication by binding to the guanine base of deoxyribonucleic acid (McGuffin et al., 2017). However, no study has been conducted to examine CFZ resistance in patients, and the MOA of CFZ in *Mab* remains unestablished. Recently, Yuanyuan et al. investigated the resistance mechanism of CFZ using 29 laboratory-induced CFZ-resistant *Mab* strains through whole-genome sequencing, wherein three genes (MAB_2299c, MAB_1483 and MAB_0540) were identified to be most commonly associated with CFZ resistance. However, future studies are needed to address the role of the identified mutations (Chen et al., 2018). CFZ shares a resistant mechanism with BDQ by up-regulating MmpL5 due to mutations in Rv0678, which is the transcriptional regulator in *Mtb.* Besides, loss-of-function mutations in *pepQ* (Rv2535c) that encode putative Xaa-Pro aminopeptidase show four times higher MICs than those for the H37Rv control in the MIC determination test with CFZ and BDQ. However, the exact mechanism by which mutations in *pepQ* provide BDQ and CFZ cross-resistance remains unclear (Andries et al., 2014; Almeida et al., 2016). Based on the BLAST similarity match, Rv0678 and PepQ (Rv2535c) have 33 and 66% amino acid sequence identity with MAB_1857c and MAB_2838c, respectively.

There are some positive *in vivo* results with CFZ for the *Mab*-infected animal model. *Drosophila* infected with *Mab* had its lifespan extended by 3 days after treatment with CFZ (Oh et al., 2014). Furthermore, Andrés et al. evaluated diverse mice infection models, such as nude (nu/nu), SCID, GKO and GM-CSF knockout mice. When GKO mice were used for *Mab in vivo* study, CFZ treatment significantly reduced bacterial burden in the lung, spleen and liver after five and 15 days of treatment. These anti-mycobacterial activities were enforced when CFZ was combined with bedaquiline (Obregón-Henao et al., 2015). Also, there are some retrospective studies of CFZ treatment for *Mab*-infected patients. Yang et al. reported a retrospective study involving 42 patients with *Mab* lung disease treated with CFZ-containing regimens between November 2013 and January 2015. In the study, CFZ achieved 24% sputum-negative culture conversion after CFZ-containing antibiotic treatment, including symptoms and radiographic improvement. Thus, the authors suggested that CFZ-containing regimens improve treatment outcomes for patients with *Mab* lung disease (Yang et al., 2017). Besides, Carey et al. also performed a retrospective cohort analysis of patients infected with *Mab* subsp. *abscessus/bolletii/massiliense*, *M. fortuitum* or *M. chelonae*. The patients were treated with a CFZ-containing regimen for over 7 years in an institution. During each treatment course, CFZ was given along with a median of five other antibiotics. Interestingly, treatment with the initial regimen was achieved in 43% of patients with pulmonary infection and 71% of patients with a nonpulmonary infection. As a component of multidrug therapy for both pulmonary and nonpulmonary RGM infections, CFZ was safe and relatively well tolerated (Carey et al., 2019).

### Inhalation Route: Nitric Oxide/Liposomal Amikacin/Molgramostim

Nitric oxide (NO) is naturally synthesised by a NO synthase in mammalian cells. No synthase activity is upregulated in the pulmonary system after infection or stimulation by cytokines in normal subjects (Xue et al., 2018). In CF patients, the increase of airway NO level results in improvement of lung function (Grasemann et al., 1997; Ho et al., 1998; Keen et al., 2010). Furthermore, NO has been shown to act as a broad-range antimicrobial agent, seen *in vitro*, *ex vivo* and animal infection models (Goldbart et al., 2020). Yaacoby-Bianu et al., previously reported that adjunctive-inhaled nitric oxide (iNO) therapy at 160 ppm for 30 min, five times/day for up to 26 days to CF patients resulted in reduced *Mab* load in sputum and improvement in pulmonary function (Yaacoby-Bianu et al., 2018). Furthermore, iNO treatment increased time to positivity in *Mycobacterium* culture with safety and tolerability albeit *Mab* culture conversion was not achieved (Bentur et al., 2020). As shown in Table 1, the iNO for patients with *Mab* has recently completed phase II clinical trials and pilot study to assess the effect of intermittent iNO on the treatment of NTM lung infection in CF and non-CF patients are currently recruiting in its clinical phase (Wu et al., 2018).

Amikacin is well known as one of the most active antibiotics for treating *Mab* pulmonary disease through the combination of one or more parenteral drugs (cefoxitin, imipenem or tigecycline) (Lee et al., 2017). Furthermore, it shows concentration-dependent bactericidal effects through irreversibly binding to the bacterial 30S ribosomal subunit, specifically in contact with 16S rRNA and S12 protein (Shi et al., 2013) (Figure 1). However, amikacin accumulates poorly in cells, which its efficacy is limited against intracellular infections (Zhang et al., 2018). The method for the targeted delivery of amikacin in the host cell is to package the antibiotic into liposomes, sphere-shaped vesicles with a membrane composed of a phospholipid bilayer (Akbarzadeh et al., 2013). The liposomes are used for drug delivery due to their unique properties, such as small size, low toxicity, tissue/cell targeting (Beltrán-Gracia et al., 2019). Based on these advantages, liposome encapsulation has been applied to amikacin to improve its killing ability in treating intracellular *Mab* infections in macrophages. When macrophages infected with the *Mab* were treated with amikacin and liposomal amikacin for inhalation (LAI), LAI at 10 mg/L showed significant effectiveness than free amikacin for intracellular *Mab* (Rose et al., 2014). This intracellular activity was further assessed in patients with treatment-refractory pulmonary NTM disease. In a clinical phase II study for *Mab-*infected patients, of four patients who achieved sputum culture conversion, three were converted after receiving LAI and one while receiving placebo at 28 days end-of-study follow-up visit. Furthermore, among the four patients who were converted, two had negative cultures 12 months after LAI discontinuation, one reverted to positive cultures, and one did not consent to participate in the 12-month follow-up phase (Olivier et al., 2017). Its efficacy and safety profiles are being further evaluated in a phase II trial against *Mab*. Currently, clinical phase II trials, especially LAI efficacy in *Mab* patients, are recruiting (Table 1).

Inhaled molgramostim is a form of GM-CSF. GM-CSF is a protein that occurs naturally in the human immune system and plays a crucial role in activating the immune system to kill bacteria. The altered immune system by molgramostim may prevent the build-up of harmful bacteria, such as NTM in the lungs (Degiacomi et al., 2019). A phase II clinical trial was conducted to test the effectiveness of inhaled mogramostim against NTM, including *Mab,* in adults with CF (Table 1). However, no *Mab*-infected patient achieved sputum culture conversion after 48-week treatment. Inversely, the participants experienced serious side effects during the treatment, and the most common adverse effect was the aggravation of bronchiectasis. The full result of inhaled molgramostim from the clinical phase II trial will be released soon (Silva, 2020).

### Gallium

Iron is essential for bacteria to mediate many key processes, such as DNA synthesis, general metabolism, electron transport and oxidative stress resistance (Andrews et al., 2003). Gallium (Ga) has a nearly identical ionic radius as iron, and some bacterial uptake systems are unable to distinguish gallium from iron. Iron is uptaken by bacterial cells, and it undergoes reduction for participation in redox cycling, which is essential for life. However, Ga cannot be reduced under normal physiological conditions within the cell, therefore, disrupting iron-dependent processes (Crunkhorn, 2018; Goss et al., 2018). For this reason, many studies have demonstrated that gallium compounds can be used as antibacterial agents against many human pathogens, including multidrug-resistant CF clinical isolates (Goss et al., 2018). Ga in the form of Ga(NO_3_)_3_ is an FDA-approved drug for treating hypercalcaemia of malignancy (Warrell et al., 1986). Recently, Abdalla et al. assessed the growth inhibitory activity of different types of Ga compound against wild-type *Mab* and clinical isolates obtained from CF and other patients. They demonstrated that Ga-protoporphyrin is the most potent type of Ga compound *in vitro* and intracellularly, and it completely inhibited the *Mabs* at much lower concentrations than Ga(NO_3_)_3_ (Abdalla et al., 2015). Furthermore, nanoparticles encapsulating Ga meso-tetraphenylporphyrine (GaTP) showed greater bactericidal activity than Ga(NO_3_)_3_ against *Mab in vitro* and intracellularly (Choi et al., 2018). Also, they evaluated the effect of drug and drug combination with Ga(NO_3_)_3_ and Ga porphyrin (GaPP) against *Mab in vitro* and in a murine pulmonary model with *Mab* infection. In this study, the authors observed that Ga(NO_3_)_3_ combined with GaPP showed significantly potent synergistic inhibitory activity against *Mab*. These findings suggest that combinations of different Ga compounds can be synergistically used for anti-*Mab* treatment in the clinic (Choi et al., 2020). Currently, Ga evaluates its safety and tolerability in adult patients with CF infected with NTM, including *Mab* as phase I (Table 1).

#### MmpL3 Inhibitors

MmpL3 is highly conserved across the mycobacteria. It is responsible for translocating mycolic acids in a trehalose monomycolate (TMM) form across the inner membrane, thus playing an essential role in cell wall synthesis (Figure 1). TMM molecules are precursors of trehalose dimycolate (TDM; cord factor) or to arabinogalactan yielding wall-bound mycolates (McNeil et al., 2020; Sarathy et al., 2020). The mmpL3 knockdown strain of *Mtb* led to a failure of cell division and, consequently rapid bacterial death due to TMM accumulation (Degiacomi et al., 2017). Recently, several MmpL3 inhibitors have been identified from the phenotypic screening of compound libraries against *Mab* and MAC. The availability of bactericidal inhibitors that target MmpL3 in *Mab* will provide opportunities to treat pulmonary *Mab* infections (Li et al., 2018).

#### PIPD1

PIPD1 is defined as a new piperidinol-based molecule. It targets the mycolic acid transporter, MmpL3, which is required to transport TMMs and abrogate the mycolylation of arabinogalactan (Dupont et al., 2016). PIPD1 shows a potential broad-spectrum *in vitro* activity against smooth (S) and rough (R) *Mab* CIP104536 with a MIC of 0.125 mg/L. The range of MBC_99_ (the concentration of the drug at which 99% of input bacilli were killed) against *Mab* S is 0.125–0.5 mg/L. In the *Mab*-zebrafish model, the treatment of infected zebrafish with PIPD1 showed increased embryo survival and decreased bacterial burden. Besides, MAB_4508 encoding a protein homologous to MmpL3 is detected as a resistant mutation to PIPD1 (Dupont et al., 2016).

#### Indole-2-Carboxamides

Indole-2-carboxamides (ICs) are another MmpL3 inhibitor and have activity against a broad spectrum of NTM pathogens. Two IC derivatives (compounds 5 and 25) prevented mycolic acid translocation from the cytoplasm to the periplasmic space by inhibiting MmpL3, consequently inducing bacterial death. Both compounds showed excellent *in vitro* activity against wild-type *Mab* ATCC 19977 (MIC of compound 5 = 0.25 mg/L and compound 25 = 0.063 mg/L) (Franz et al., 2017). Compounds 25- and 5-resistant mutant harbours a missense mutation (A309P) in the MmpL3 protein and this mutant showed 16–128-fold increase in MICs of compounds 25 and 5, respectively (MIC of compound 5 against the A309P missense mutant = 32 mg/L; MIC of compound 25 against the A309P mutant = 1 mg/L) (Pandya et al., 2019). Both derivatives showed statistically significant reduced bacterial load in the lung and spleen of Mab-infected mice in the acute SCID treatment mouse model. This *in vivo* effectiveness of ICs compounds was similar to amikacin used as a positive control (Pandya et al., 2019). The sequencing result of the MmpL3 gene from laboratory-induced resistant mutants against high-dose PIPD1 or on indole-2-carboxamides revealed a common Ala309Pro substitution in MmpL3. Additionally, the overexpression of MmpL3 carrying the Ala309Pro mutation in *Mab* showed high-level resistance to PIPD1 and indole-2-carboxamides (Dupont et al., 2016; Kozikowski et al., 2017).

In other studies with IC analogues by Kozikowski et al., lead compounds 6 and 12, which have favourable absorption, distribution, metabolism and excretion properties, also exhibited strongly *in vitro* activity against *Mab* clinical isolates from CF and non-CF patients and especially compound 12 showed significantly reduced number of *Mab*-infected cells. The biochemical assay revealed that treatment of compound 12 strongly accumulates TMM on thin-layer chromatography, resulting in the defect of trehalose dimycolate production (TDM) synthesis, consequently failure of mycolylation of arabinogalactan (Kozikowski et al., 2017). These compounds 6 and 12 were further studied in combinations with imipenem and cefoxitin *in vitro* by checkerboard assay to evaluate their synergistic effect against *Mab*. Raynaud et al.*,* demonstrated that combination between IC (compounds 6 and 12) plays a synergistic role with imipenem and cefoxitin *in vitro* and, especially compound 12 also showed synergistic effect with imipenem in THP-1 macrophages. This potential synergistic effect requires further pre-clinical animal study (Raynaud et al., 2020b).

#### EJMCh-6

EJMCh-6, which is a benzimidazole analogue targeting MmpL3 in *Mab*, shows a potential broad-spectrum *in vitro* activity against smooth (S) *Mab* CIP104536^T^ with a MIC of 0.125 mg/L. EJMCh-6 is bacteriostatic *in vitro* and this compound showed potent activity with MIC values ranging from 0.031 to 1 mg/L against the various strains and subspecies of *Mab* that were isolated from patients with or without CF. In the THP-1 cell model of infection with *Mab*, EJMCh-6 exerted a very strong activity against intramacrophage-residing *Mab*. In the *Mab*-zebrafish model, approximately 80% of the treated embryos survive at 12 dpi after being treated with 0.75 mg/L EJMCh-6 (Raynaud et al., 2020a).

#### Benzothiazole Amide Compounds

Benzothiazole Amide Compounds (CRS400226, CRS400153, CRS400359 and CRS400393) target MmpL3 and further showed excellent *in vitro* activity against *Mab* infections with an MIC_90_ of 0.5 mg/L. Among those analogues, Mary *et al.* assessed the *in vivo* efficacy of CRS400226 against *Mab* in chronic lung infection using GM-CSF mice for 28 days. Intratracheal administration of CRS400226 at 25 mg/kg/day for 28 days resulted in 0.64 log_10_ CFU reduction compared with the vehicle control. The histological assay revealed that CRS400226-treated animals had only minor areas of airway-centric inflammation (De Groote et al., 2018).

### Newly Discovered Anti-*Mab* Inhibitors

#### Leucyl-tRNA Synthetase Inhibitor (Epetraborole and Benzoxaborole EC/11770)

Epetraborole (also known as AN 3365; GSK 2251052) is a benzoxaborole analogue and leucyl-tRNA synthetase inhibitor (O’Dwyer et al., 2015; Purnapatre et al., 2018). It has shown a novel mode of action against gram-negative bacterial infections, such as urinary tract infections. In previous studies, the benzoxaborole analogue showed antitubercular activity against *Mtb* and *M. smegmatis* (Palencia et al., 2016). Recently, Kim et al. performed *in vitro* dual-screen against *Mab* R and S variants using a pandemic response box that comprises 400 structurally diverse compounds (201 antibacterials, 153 antivirals and 46 antifungals) and is a drug library assembled by the Medicines for Malaria Venture (MMV) (Kim et al., 2021). Through the screen, the authors identified epetraborole, which showed significant activity against *Mab* wild-type strain growth in three subspecies, drug-resistant strains, clinical isolates *in vitro* (MIC_90_ ranges from 0.1 to 1.35 mg/L), intracellular and in the zebrafish infection model. However, according to ClinicalTrials.gov in 2017, a clinical phase II study of epetraborole for treating complicated urinary tract infection and intra-abdominal infection was terminated due to the rapid emergence of drug resistance during treatment (Purnapatre et al., 2018). Recently, another benzoxaborole analogue named benzoxaborole EC/11770 was also identified against *Mab* by testing advanced compounds that already showed activity against *Mtb*. The EC/11770 showed antibacterial activity against *Mab* and *M. avium* complexes. Furthermore, EC/11770 inhibited the growth of the *Mab* biofilm *in vitro* model and showed effective *in vivo* efficacy in the NOD SCID mice lung infection model. Fortunately, EC/11770 showed low-resistant mutant frequency, and leucyl-tRNA synthetase was confirmed as its target (Ganapathy et al., 2021).

#### AR-12 (OSU-03012)

AR-12 (OSU-03012) is a novel derivative of celecoxib that inhibits phosphoinositide-dependent kinase-1 (PDK1) activity in different cell models and has further progressed to phase I clinical trial as an anticancer agent. Intriguingly, it exhibits anti-pathogenic activity against bacteria, fungi and viruses. According to mechanistic studies, AR-12 represses the host cell chaperone machinery, thus preventing proper viral protein folding and effective viral assembly. AR-12 also causes autophagy, which aids in clearing intracellular viruses, unfolded proteins or both (Abdulrahman et al., 2017). In the aspect of *Mab* activity, AR-12 displayed broad anti-*Mab* activity against 194 clinical *Mab* isolates (148 subsp. *abscessus* and 46 subsp. *massiliense*) with a moderate MIC value (MIC_90_ of 4 and 8 mg/L). Furthermore, AR-12 showed growth inhibitory activity against *Mab* residing within primary peritoneal macrophages. Lastly, AR-12 inhibited *Mab* replication in a mouse model with a lung infection. AR-12 (50 mg/kg) caused significant reductions of approximately 3.7 log_10_ CFU in the lung after 2 weeks of treatment. The histological assay also revealed a reduction in inflammatory pathology and bacterial counts in the lungs of AR-12-treated mice compared with untreated mice (Zhang et al., 2020).

#### SPR719

SPR719 (previously VXc-486) is a novel aminobenzimidazole that targets ATPase subunits (GyrB/ParE) in *Mtb* (Locher et al., 2015). It is a novel class that targets the ATPase subunits of gyrase by a mechanism distinct from fluoroquinolones (Verma et al., 2020). The MIC_90_ value for subspecies *Mab* subsp. *abscessus*, *M ab* subsp. *massiliense* and *M. abscessus/massiliense* hybrid was 2–4 mg/L. Although *Mab* contains a natural A92S mutation in its *gyrB*, the potency of SPR719 against *Mab* in the MIC test was greater than that of moxifloxacin, which was used as a control (Locher et al., 2015). Recently, Rubio et al. (2018) reported the *in vivo* efficacy result of SPR720 (a prodrug of SPR719) using a SCID mice model of infection with *Mab* subsp. *bolletii* (strain 103) at ASM Microbe 2018. Treatment was administered via oral dosing (25, 50, 100, 200, 300 and 400 mg/kg/day). After 16 days of treatment, the bacterial burden in the lung, spleen and liver was enumerated. From the study, a daily dosage of 100 mg/kg demonstrated the most significant bacterial reduction in the lung, spleen and liver compared with that in the control group (Rubio A, Stapleton M, Verman D).

#### Etamycin

Etamycin (also called viridogrisein) is a cyclic peptide antibiotic isolate of marine actinomycete. Recently, etamycin showed potent activity against wild-type *Mab*, three subspecies of the *Mab* complex and clinical isolates, including the R and S variant, at an MIC_50_ level of 1.6–7.2 mg/L. Furthermore, etamycin inhibited the growth of *Mab* that resides in macrophages without cytotoxicity. The *in vivo* efficacy of etamycin in the zebrafish infection model was greater than that of clarithromycin. ZF’s survival rate of 44 mg/L (50 μM) etamycin treatment was 85%, higher than that observed for the treatment with 37.4 mg/L (50 μM) clarithromycin at 13 days post-infection (Hanh et al., 2020a).

#### Thiostrepton

Thiostrepton is an FDA-approved antimicrobial drug for animal use and is a quinaldic acid moiety containing a natural thiopeptide. It has been remarkably determined to be an effective translational blocker that binds to nucleotides A1065 and A1095 on helices 43 and 44 of the 23S rRNA. Also, it binds proline residues within the N-terminal domain of uL11 that is a ribosomal protein. Thiostrepton has antibacterial activity against *Staphylococcus aureus* (MRSA), methicillin-resistant *Enterococcus faecium*, penicillin-resistant *Streptococcus pneumoniae*, vancomycin-resistant enterococci, *Mtb* and *M. marinum*. Thiostrepton significantly inhibited the growth of *Mab in vitro* and macrophage-infected *Mab*. Furthermore, thiostrepton significantly decreased proinflammatory cytokine production in macrophages, suggesting an inhibitory effect of thiostrepton on inflammation-induced during *Mab* infection. Also, thiostrepton exhibits antimicrobial effects *in vivo* in zebrafish models of *Mab* infection (Kim et al., 2019). Nevertheless, thiostrepton has limitations for its clinical use. It has a large molecular size, lacking bioavailability and poor aqueous solubility (Just-Baringo et al., 2014). Recently, newly developed semisynthetic thiopeptide, LFF571, by Novartis showed an improved pharmacokinetic profile, such as aqueous solubility, compared to thiostrepton. LFF571 tested for *Clostridium difficile*, which causes intestinal infections in humans. LFF571 showed excellent efficacy in a hamster model with a lower dose and fewer recurrences than vancomycin (Trzasko et al., 2012).

## Conclusion

*Mab* is described as an environmentally derived opportunistic pathogen that likely causes lung and skin infections in patients with a weak immune system in various environments. *Mab* infection can be acquired when taking a shower from the showerhead and during cosmetic surgery and acupuncture. Therefore, it is currently essential to proactively identify the potential severity of *Mab* infection in communities and healthcare institutions. It is also important to re-evaluate the case of *Mab* misdiagnosed as TB to determine the current infection situation of *Mab* accurately. Presently, the most crucial aspect of treating *Mab* is the formation of a sputum smear transition quickly. As mentioned above, various antibiotics, including anti-TB drugs, are currently used to treat *Mab* through combination therapy, but only a few drugs can derive sputum smear conversion. Therefore, developing new drug recombination using various antibiotics, including newly identified anti-TB drugs and various efflux pump inhibitors, may be a priority.

Next, the development of new antibiotics using new libraries would be essential. Lipinski’s rule-of-five (Ro5: not more than five and one hydrogen bond donors and hydrogen bond acceptors, respectively, and a partition coefficient (log P) value less than 5) has been used for selecting candidates with good oral bioavailability properties. However, the Ro5 sometimes cannot explain the bioactivity of natural products that systematically break the Ro5 with bioavailability and bioactivity. Thus, screening natural products with expanded chemical diversity for discovering new drugs would be an alternative step for drug development against *Mab*. Natural compounds with bioavailability and bioactivity that can penetrate membranes of host cells and kill intracellular *Mab* would be new weapons that will expand the success rate in a drug screen. Drug screen model systems for excavating these substances are also very crucial. Developing new drugs for *Mab* through a new screen model that mimic the human environment is needed.

Investing in pharmaceutical companies for *Mab* is currently passive. This is probably because there are fewer economic benefits for chronic diseases, such as cancer, diabetes, and degenerative neurological and brain diseases. Therefore, research in small- and medium-sized enterprises and universities is currently leading to a small number of studies on *Mab* infections and antibiotics. However, a report by the United Kingdom government says the number of deaths from antibiotic-resistant bacteria by 2050 will far outweigh the number of deaths caused by chronic diseases, such as cancer, diabetes and diarrhoeal disease. Without antibiotics, we cannot perform surgery on premature infants, treat diseases like cancer and perform organ transplants and plastic surgery. Lung disease caused by *Mab* infection is steadily increasing worldwide; and due to an increase in the population of elderly individuals through an ageing society, and the increase in long-term care patients, *Mab* infection is seen to be a global public health concern. Therefore, developing new drugs for *Mab*, which minimises side effects, is a challenge that must be undertaken. Significant investment must be made to develop new drugs and fundamental research on *Mab*, which is resistant to various antibiotics. Thus, this will lead to the screening of *Mab*-effective drugs in clinical trials.
